# Machine learning-driven survival prediction in gestational trophoblastic neoplasms: a focus on PSTT and ETT prognosis

**DOI:** 10.3389/fonc.2024.1457531

**Published:** 2024-09-30

**Authors:** Sakhr Alshwayyat, Zena Haddadin, Mustafa Alshwayyat, Tala Abdulsalam Alshwayyat, Ramez M. Odat, Mohammed Al-mahdi Al-kurdi, Saoussane Kharmoum

**Affiliations:** ^1^ Faculty of Medicine, Jordan University of Science & Technology, Irbid, Jordan; ^2^ Faculty of Medicine, University of Aleppo, Aleppo, Syria; ^3^ Médical Oncology, Régional Hospital Center, Tangier, Morocco

**Keywords:** clinical decision making, machine learning, placenta, prognosis, survival analysis, treatment outcome, trophoblastic tumor

## Abstract

**Introduction:**

The clinicopathological characteristics and prognosis of placental site trophoblastic tumor (PSTT) and epithelioid trophoblastic tumor (ETT) have not been well summarized. Consequently, we conducted the largest to date series of samples of both types and employed machine learning (ML) to assess treatment effectiveness and develop accurate prognostic models for patients with GTN. Gestational choriocarcinoma (GCC) was used as the control group to show the clinical features of PTSS and ETT.

**Methods:**

The Surveillance, Epidemiology, and End Results (SEER) database provided the data used for this study’s analysis. To identify the prognostic variables, we conducted Cox regression analysis and constructed prognostic models using five ML algorithms to predict the 5-year survival. A validation method incorporating the area under the curve (AUC) of the receiver operating characteristic (ROC) curve was used to validate the accuracy and reliability of the ML models. We also investigated the role of multiple therapeutic options using the Kaplan-Meier survival analysis.

**Results:**

The study population comprised 725 patients. Among them, 139 patients had ETT, 107 had PSTT, and 479 had GCC. There were no significant differences in survival between the different tumor groups. Multivariate Cox regression analysis revealed that metastasis was a significant prognostic factor for GCC, while older age and radiotherapy were significant prognostic factors for PTSS and ETT. ML models revealed that the Gradient Boosting classifier accurately predicted the outcomes, followed by the random forest classifier, K-Nearest Neighbors, Logistic Regression, and multilayer perceptron models. The most significant contributing factors were tumor size, year of diagnosis, age, and race.

**Discussion:**

Our study provides a method for treatment and prognostic assessment of patients with GTN. The ML we developed can be used as a convenient individualized tool to facilitate clinical decision making.

## Graphical Abstract

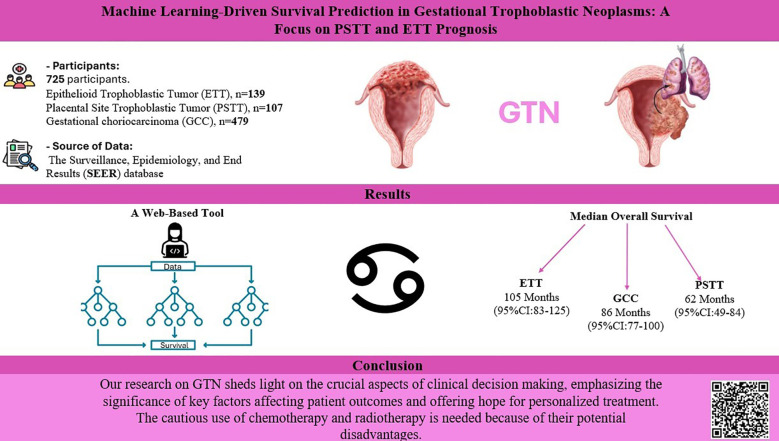

## 1 Introduction

Gestational trophoblastic neoplasms (GTNs) are a group of malignant tumors originating from placental villous and extra-villous trophoblasts, consisting of invasive mole, choriocarcinoma, placental site trophoblastic tumor (PSTT), and epithelioid trophoblastic tumor (ETT) (1–3). While most patients are asymptomatic, vaginal bleeding is the most common presenting symptom (4).

PSTT is the least common form of GTN and originates from extravillous intermediate trophoblasts on the maternal side of the placental bed that invade the myometrium. The average age at the presentation is 31 years, ranging from 20 to 63 years (4, 5). Abnormal vaginal bleeding (31.3-79.4% of cases) is the most common symptom, followed by amenorrhea (5, 6).

ETT originates from chorionic laeve-type intermediate trophoblasts and can be found in the uterus (40%), uterine cervix (31%), or extrauterine sites, such as the lungs (19%), fallopian tubes, ovaries, and pelvic peritoneum (6, 7). The mean age of women affected by ETT is 36.1 years, 15–48 years) (5). Abnormal vaginal bleeding (57-67% of cases) is the most common symptom, followed by amenorrhea, abdominal pain, and abdominal bloating (6).

Contrary to widespread belief, hCG is not an accurate marker of PSTT/ETT disease because both tumors do not secrete hCG due to their origin from intermediate trophoblasts instead of syncytiotrophoblasts (3, 5, 8).

The primary treatment for PSTT and ETT is surgical intervention, including hysterectomy and resection of the metastatic disease. Hysterectomy has a good survival rate for stage I ETT and PSTT, with over 90% of patients surviving for ten years. Conservative options, such as uterine curettage and hysteroscopic resection, may be considered for fertility preservation. In some cases, chemotherapy may be administered in addition to surgery. However, ETT is generally resistant to conventional chemotherapies. Surgical resection and chemotherapy have led to 10-year overall survival rates of approximately 50% for stage II-IV disease (1, 9).

Artificial intelligence (AI) includes ML, which focuses on computer data analysis and learning algorithm refinement. ML has been successful in addressing complex problems, especially in medicine, where it has been applied to medical image recognition, treatment support, and biomedical research (10–12).

Owing to the scarcity of cases of PSTT and ETT, their clinicopathological characteristics and prognosis have not been well summarized. Additionally, it is crucial to develop reliable predictions for personalized care and improved management. In this study, we used ML techniques to predict survival and identify potential prognostic factors. Because GCC is the most common type among GTN, we set it as a control group to show the clinical features of PSTT and ETT.

## 2 Materials and methods

### 2.1 Data extraction and variables

**Figure 1:**
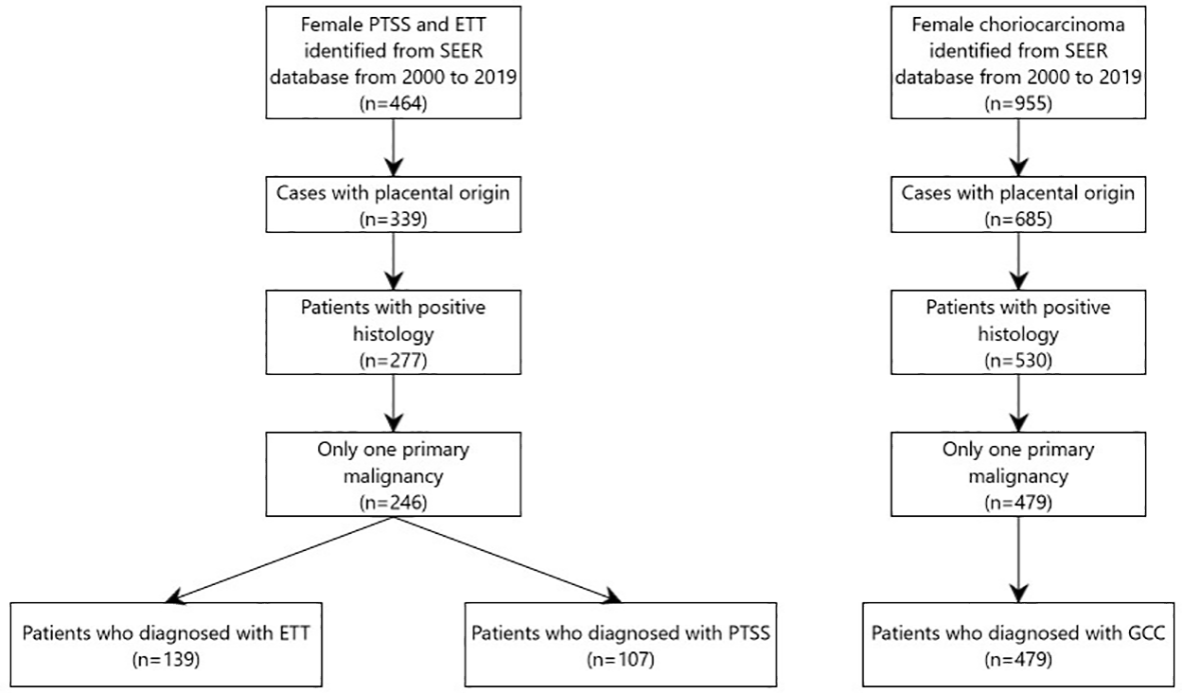
Flow diagram for patients’ selection.

We extracted the data from “Incidence-SEER 18 Regs Custom Data (with additional treatment fields), Nov 2020 Sub (2000–2019 varying)” database. All patients diagnosed between 2000 to 2019 were identified using SEER*Stat software (version 8.4.0.1). Data of all patients diagnosed based on the third edition of the International Classification of Diseases for Oncology (ICD-O-3) were included in this study. Histology codes for disorders were as follows: (i) GCC included 9100/3 (choriocarcinoma); (ii) PTSS: 9104/3 (malignant placental site trophoblastic tumor); and (iii) ETT: 9105/3 (trophoblastic tumor, epithelioid). Patients who met any of the following criteria were excluded: diagnosis not confirmed by histology, not the first tumor or other malignancies in the body, and not arising from the placenta. Finally, 139 patients were eligible for ETT, 107 for PTSS, and 479 for GCC. The detailed screening process is shown in the flow diagram in (**Figure 1**).

The following SEER variables were selected for our study: Patient characteristics (including age, race, and marital status), tumor characteristics (including SEER stage, tumor size and metastasis), therapeutic methods (including surgery, chemotherapy, and radiotherapy), and survival information were obtained from the database. Age was divided into two groups: ≤30 and >30 years; SEER stages were classified into two groups: localized (within the organ), regional (extension to adjacent organs or regional lymph nodes) and distant (involvement of distant organs or distant metastasis). The primary survival outcomes were overall survival (OS) which was calculated from diagnosis to any cause of death and cancer-specific survival (CSS) which was calculated from diagnosis to death caused by GTN.

### 2.2 Statistical analysis

#### 2.2.1 Data processing and statistical methods

The All data were analyzed using R software (v4.0.0) and the following packages: “readxl,” “tidyverse,” “Hmisc,” “data.table,” “**Table 1**,” “MatchIt,” “survminer,” “survival,” and “broom.” Chi-square analysis was used to assess categorical variables and evaluate the clinicopathological characteristics of patients with GTN. The K-M method was used to estimate survival rates, and the log-rank test was used to analyze differences between survival curves. Multivariate Cox proportional hazards models identified independent predictors of OS and CSS. A two-tailed P value less than 0.05 was considered statistically significant. Five ML ensembles were employed: random forest classifier (RFC), Gradient Boosting classifier (GBC), Logistic Regression (LR), K-Nearest Neighbors (KNN), and multilayer perceptron (MLP). These algorithms were selected because of their ability to handle complex multidimensional datasets. All ML implementations were processed using the scikit-learn 0.18 package in Python.

**Table 1:** Clinicopathological characteristics.

Category Characteristics	ETT (N=139)	GCC (N=479)	PSTT (N=107)	Overall (N=725)	P-value
Age					
< 30 years	53 (38.1%)	226 (47.2%)	42 (39.3%)	321 (44.3%)	0.182
> 30 years	86 (61.9%)	253 (52.8%)	65 (60.7%)	404 (55.7%)	
Race					
Black	40 (28.8%)	102 (21.3%)	19 (17.8%)	161 (22.2%)	<0.001
Other	10 (7.2%)	71 (14.8%)	31 (29.0%)	112 (15.4%)	
White	89 (64.0%)	306 (63.9%)	57 (53.3%)	452 (62.3%)	
Year of diagnosis					
2000-2009	93 (66.9%)	272 (56.8%)	47 (43.9%)	412 (56.8%)	0.005
2010-2019	46 (33.1%)	207 (43.2%)	60 (56.1%)	313 (43.2%)	
Marital status					
Married	66 (47.5%)	256 (53.4%)	60 (56.1%)	382 (52.7%)	0.549
Not married	73 (52.5%)	223 (46.6%)	47 (43.9%)	343 (47.3%)	
Stage					
Distant	64 (46.0%)	205 (42.8%)	61 (57.0%)	330 (45.5%)	<0.001
Localized	46 (33.1%)	208 (43.4%)	22 (20.6%)	276 (38.1%)	
Regional	29 (20.9%)	66 (13.8%)	24 (22.4%)	119 (16.4%)	
Tumor size					
< 4 cm	73 (52.5%)	45 (9.4%)	48 (44.9%)	166 (22.9%)	<0.001
> 4 cm	66 (47.5%)	434 (90.6%)	59 (55.1%)	559 (77.1%)	
Metastasis					
No	74 (53.2%)	274 (57.2%)	43 (40.2%)	391 (53.9%)	0.017
Yes	65 (46.8%)	205 (42.8%)	64 (59.8%)	334 (46.1%)	
Surgery of the primary tumor					
Not performed	53 (38.1%)	211 (44.1%)	41 (38.3%)	305 (42.1%)	0.517
Surgery performed	86 (61.9%)	268 (55.9%)	66 (61.7%)	420 (57.9%)	
Chemotherapy					
No	42 (30.2%)	105 (21.9%)	28 (26.2%)	175 (24.1%)	0.228
Yes	97 (69.8%)	374 (78.1%)	79 (73.8%)	550 (75.9%)	
Radiotherapy					
No	136 (97.8%)	460 (96.0%)	98 (91.6%)	694 (95.7%)	0.107
Yes	3 (2.2%)	19 (4.0%)	9 (8.4%)	31 (4.3%)	

#### 2.2.2 Model training

To ensure model robustness, standard scaling was applied uniformly across all features using the StandardScaler module from Scikit-learn. This normalized the data, thereby alleviating the potential impacts of different scales among the features. Subsequently, ML models were trained on a dataset with a binary classification output predicting the target “5-year survival.” The features included demographics, tumor characteristics, and management approaches. The dataset was randomly split into 7:3 for training (n =172) and testing (n =74) sets for the PSTT and ETT. In addition, training (n =335) and testing (n =144) sets were used for GCC.

#### 2.2.3 Feature importance and model evaluation

The feature contribution in predicting “5-year survival” was calculated using the permutation importance method. ROC curves and AUC scores were used to evaluate the model discriminatory power. The roc_curve function from the sklearn.metrics module computes false positive rate (FPR) and true positive rate (TPR). The predicted probabilities of the positive class were obtained using the predict-proba method for each model. AUC scores were calculated using the roc_auc_score function. A custom plotting function, plot_roc_curve, and visualized ROC curves of the multiple models. Additional model evaluation included a mean bootstrap estimate with a 95% Confidence Interval, 10-fold cross-validation, and a classification report for precision, recall, and F1-score.

## 3 Results

### 3.1 Patient characteristics

A total of 728 patients who met the inclusion criteria were included in the study (**Table 1**). The median age at diagnosis was 37 years. Most of the patients were of white race (62.3%) and more than half were married (52.7%). The most common stage of primary tumor histology was distant metastasis, which occurred in 330 patients. The average tumor size was 6.13 cm. Notably, 46.1% of the patients had a primary tumor that had already metastasized. More than two-thirds of the patients underwent chemotherapy (75.9%), 57.9% underwent surgery, and 4.3% underwent radiotherapy. Compared to the GCC group, the ETT and PSTT groups had significantly more patients with smaller primary tumor sizes. The highest rate of metastatic primary tumors was observed in the PSTT group (59.8% vs. < 47% in ETT/GCC). The rate of primary surgical resection was highest in the ETT patient group, followed by the PSTT and GCC groups, whereas there was no significant difference in the rates of receiving chemotherapy and radiotherapy.

### 3.2 Survival analysis between gestational trophoblastic tumors

**Figure 2:**
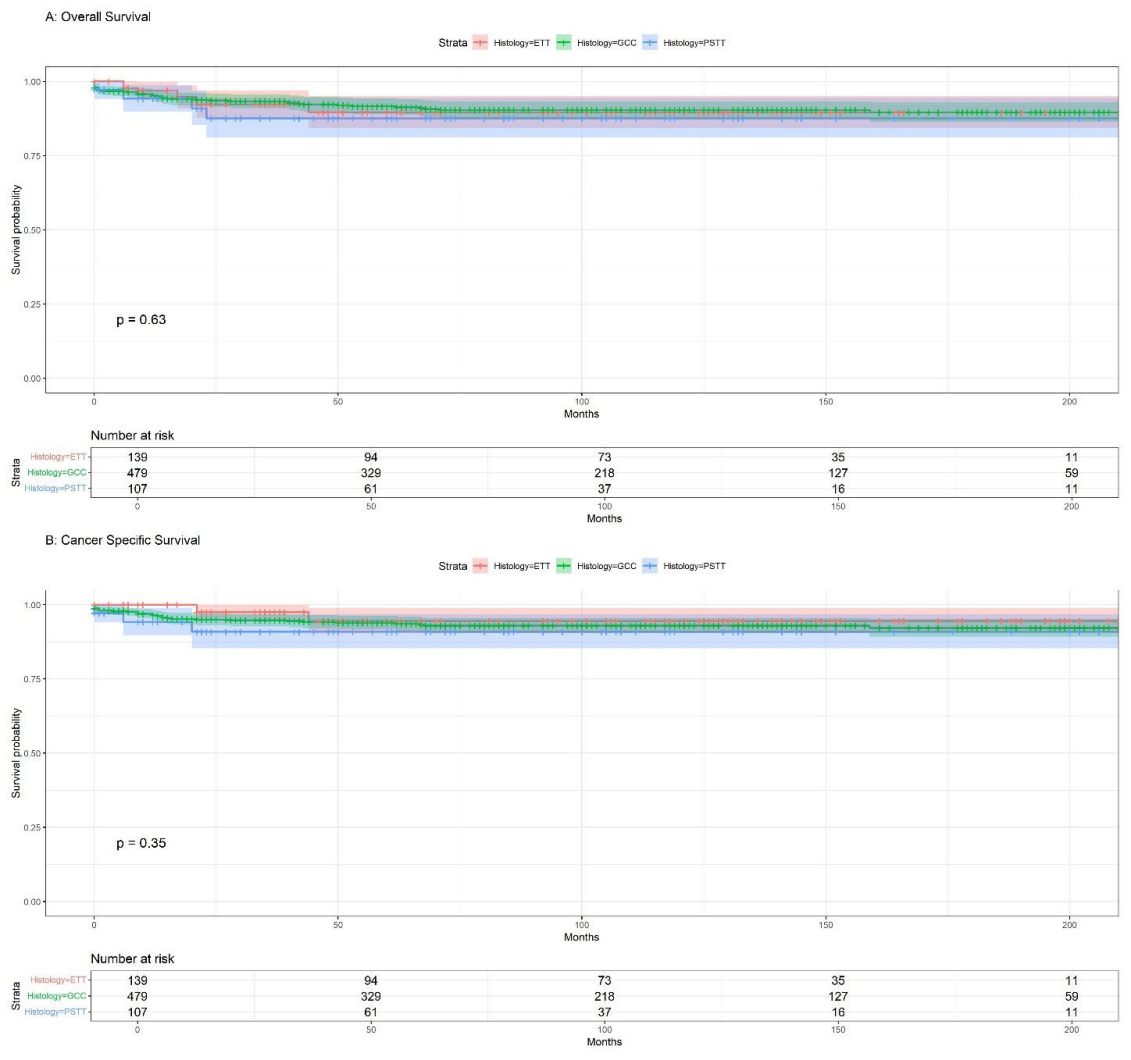
**(A)** Kaplan-Meier overall survival (OS) for GTN; **(B)** Kaplan-Meier cancer-specific survival (CSS) for GTN.

Survival analysis was performed to investigate potential differences in OS and CSS among the three groups, as shown in (**Figure 2**). The results did not indicate statistically significant differences in OS or CSS between the groups (p=0.63, p=0.35, respectively). The median overall survival for the ETT group was 105 months (95%CI:83-125), 86 months (95%CI:77-100) for the GCC group, and 62 months(95%CI:49-84) for the PSTT group, and the difference was compared using the Kruskal-Wallis test(p=0.05005). Regarding the median CSS, the differences between the groups were not statistically significant (p=0.395). Although statistically insignificant, GCC presented the highest 1-, 3-, and 5-year overall survival rates among all GTN groups (95%, 93%, and 92%, respectively), whereas ETT showed the highest 1-, 3-, and 5-year cancer-specific survival rates, albeit statistically insignificant (**Supplementary Table S1**).

### 3.3 Prognostic factors

**Figure 3:**
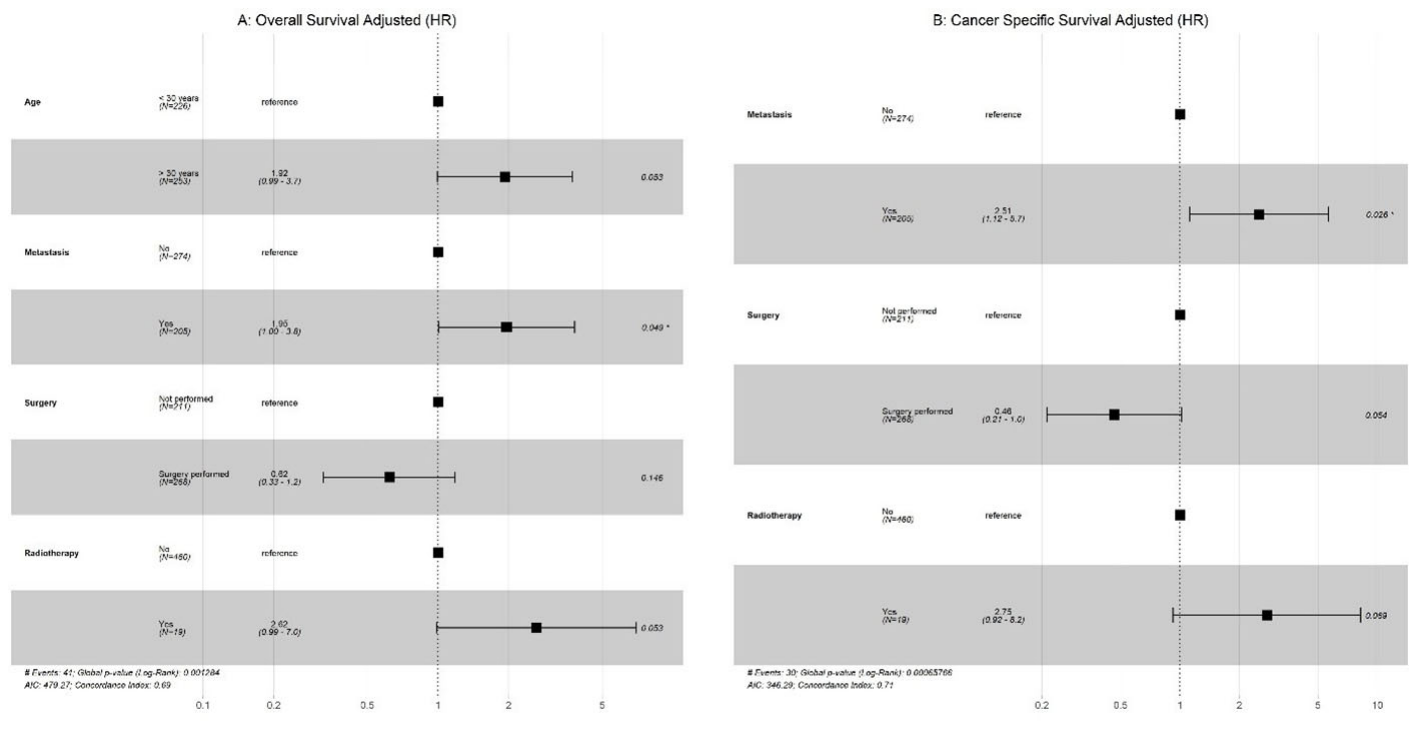
GCC: **(A)** Multivariate Cox regression for overall survival; **(B)** Multivariate Cox regression for cancer-specific survival (CSS).

**Figure 4:**
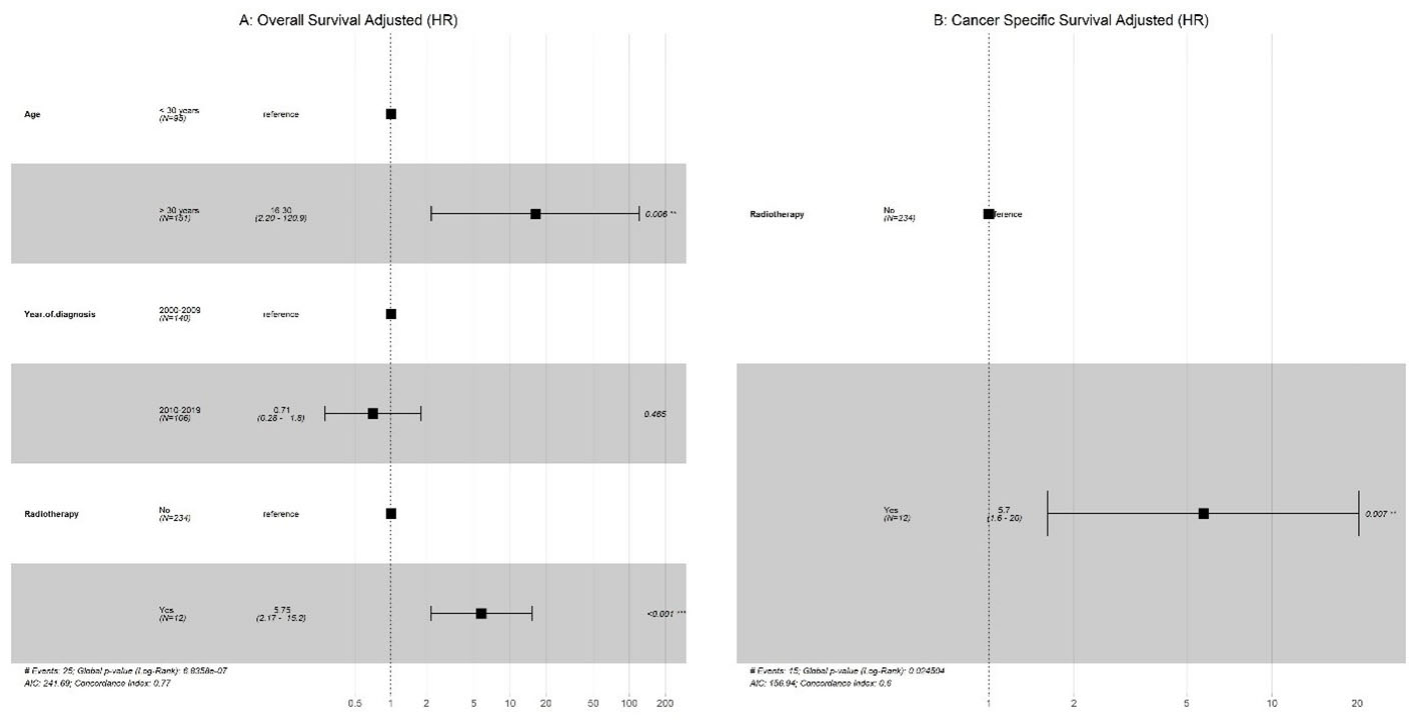
PTSS + EST: **(A)** Multivariate Cox regression for overall survival; **(B)** Multivariate Cox regression for cancer-specific survival (CSS).

Univariate analysis was performed, and only significant variables were considered for the multivariate Cox regression. As shown in (**Figure 3** **),** our results indicated that metastasis had a significantly poor effect on OS and CSS in patients with GCC. Radiotherapy had a significant effect on OS and CSS, whereas older age only on OS in patients with PSTT and ETT (**Figure 4**).

### 3.4 Model performances and interpretability

**Figure 5:**
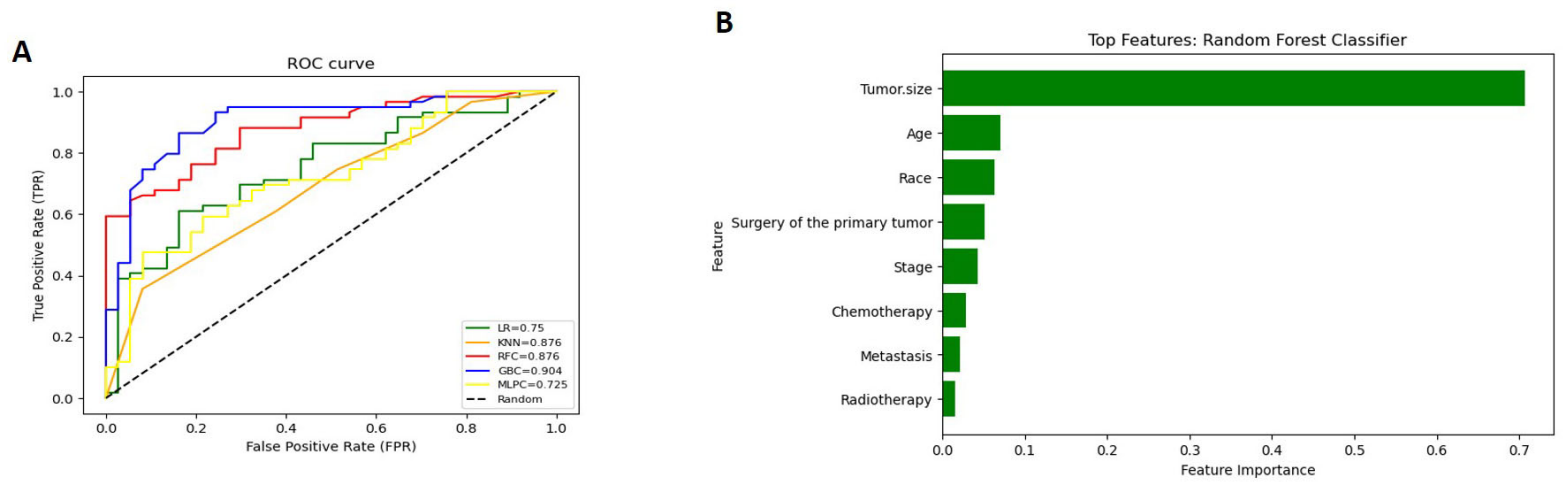
(for GCC): **(A)** Receiver Operating Characteristic (ROC) curves of all Machine Learning Models (MLMs); **(B)** Permutation Features Importance (Random Forest Classifier).

**Figure 6:**
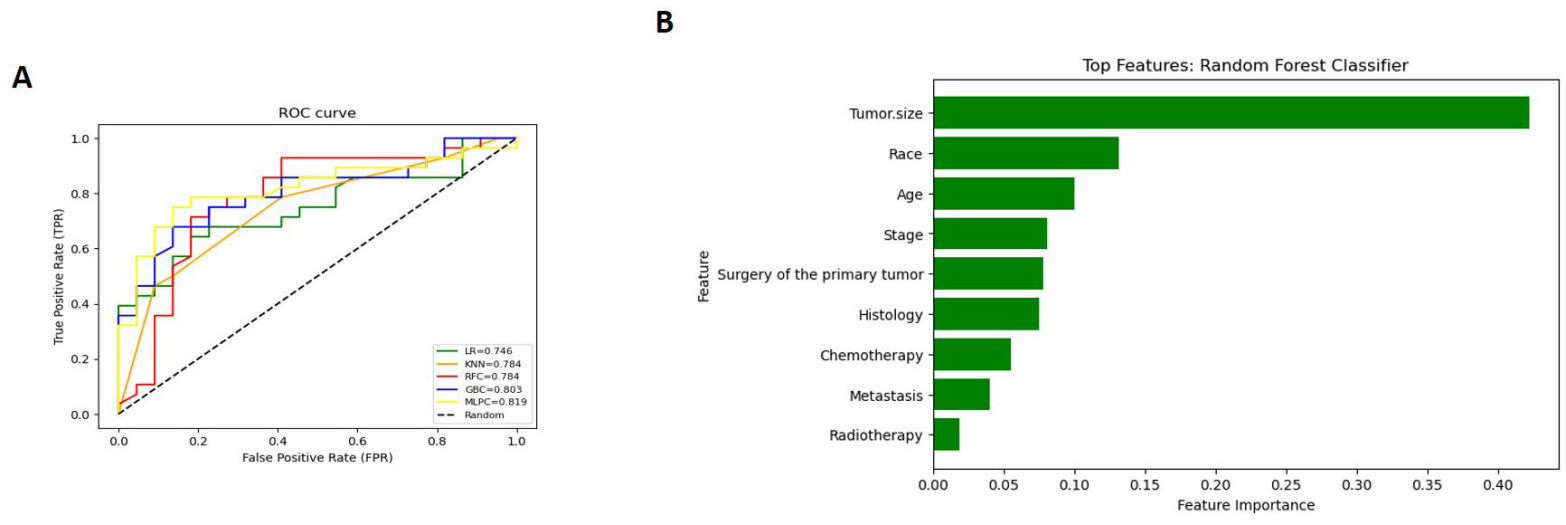
(for ETT and PTSS): **(A)** Receiver Operating Characteristic (ROC) curves of all Machine Learning Models (MLMs); **(B)** Permutation Features Importance (Random Forest Classifier).

The Detailed performance metrics for all machine learning algorithms (MLMs) are summarized in (**Supplementary Table 3**). ROC curves of all MLMs and the most contributing features in prediction in the RFC model are displayed in (**Figure 5**) for GCC and in (**Figure 6**) for PTSS and ETT. A web-based tool was developed using a random forest model that incorporated various clinical variables to predict the survival of patients diagnosed with GTN. Clinicians and researchers can input patient-specific data, such as age, sex, race, tumor stage, tumor size, treatment type, and histology, to estimate survival duration.

The interactive tool, designed for ease of use and rapid calculation, is accessible through the following link: https://sakhrshwayyat.shinyapps.io/Gestational_Trophoblastic_Neoplasia/. For instance, upon entering patient data, the tool instantly predicts the expected survival months.

## 4 Discussion

PSTT and ETT are uncommon forms of GTN originating from intermediate trophoblast cells. Because their origin differs from other GTN types, it is expected that the clinical presentation of PSTT and ETT, their tumor marker profile, and their treatment approach also differ significantly (3). Our research examined various factors to determine their relationship with survival, and we were the first to develop AI prognostic models for GTN patients, which have proven to be highly accurate in predicting the survival of these patients.

Based on our research, being older (age 30 or above) is linked to a less favorable outcome in terms of both OS and CSS. This aligns with certain sources, indicating that advanced age is a risk factor for a poor prognosis in GTN (1, 13, 14). However, other studies have suggested that age does not significantly affect survival and does not necessarily result in poor outcomes (15, 16).

After reviewing the literature, a study conducted in the UK screened all PSTT/ETT diagnoses between 1973 and 2014. Its prevalence was 0.2%, with a mortality rate of 19% (14). In contrast, another study in China retrospectively analyzed 108 PSTT patients registered in two GTD centers or six tertiary hospitals between 1998 and 2013, revealing an incidence of 3% and a mortality rate of only 6.5% (1). The differences between these findings in China and the UK could be attributed to the Chinese study focusing solely on patients with PTSS, unlike the UK study, which included both PSTT and ETT cases. Additionally, differences in population genetics, potentially due to distinct racial backgrounds and variations in management approaches, might contribute to these differences (17). Although about 60% of the patients in our study were white, race did not significantly affect prognosis, as shown through our Cox regression analysis. However, ML models revealed that race was one of the significant factors affecting survival, indicating the need for further research.

In the literature, suggesting that tumor size is not a prognostic factor (6, 18). Nevertheless, conflicting viewpoints exist in a few studies that argue that tumor size is associated with a poorer prognosis (17, 19).

The Cox regression results of our study indicated that tumor size does not play a significant role in the OS and CSS of patients with ETT and PSTT. However, our ML models revealed that tumor size plays an important role in survival prediction, which may further reflect the superiority of ML. Specifically, unlike traditional linear regression analysis, which has the problem of overfitting, it can better eliminate unnecessary features. ML enables us to obtain more accurate predictive models by continuously improving operational efficiency and self-improvement.

Multiple studies have highlighted the significance of patient cancer staging (FIGO classification) as a crucial prognostic indicator (1, 13, 18, 20). One study conducted on patients with PSTT at a tertiary care center between 1996 and 2011 emphasized that stage IV disease stood out as a highly significant and clinically relevant predictor of poor prognosis (15). Another study, involving patients sourced from the International PSTT and ETT database, found that patients with FIGO stages II–IV exhibited worse outcomes than those with FIGO stage I disease, based on univariable analysis (18). However, our study lacked FIGO stage information but included SEER stage and metastasis data. In addition, our study results indicated that presence of metastasis disease significantly impacts survival months.

In our research, approximately 58% of the patients underwent surgery for the primary tumor. The usual indication for surgery in the early stages, often serving as the primary treatment in stage I, where the disease is less advanced and in its early phase. Numerous studies in the literature advocate surgery as the initial management for both ETT and PSTT (1, 18, 20). Several surgical procedures have been mentioned in the literature, including total abdominal hysterectomy, endometrial resection, excision of extrauterine lesions, pelvic lymph node dissections, curettage, and excision of uterine lesions (1, 20). While hysterectomy is commonly recommended, conservative surgery may be considered for young patients desiring fertility preservation (21). In premenopausal women, the standard practice involves preserving the ovaries unless the disease is evident or there is a family history of ovarian cancer (20). For other forms of GTN, surgical resection of residual masses does not offer any benefits (22). However, a study involving 62 patients with PTSS revealed the importance of resecting residual masses after detecting viable tumor cells in some patients undergoing mass resection. This was explained by the lower sensitivity of PSTT to chemotherapy (20).

PSTT and ETT have a slower cell growth rate than other types of GTN. Therefore, they do not respond as strongly to chemotherapy, distinguishing their treatment approach from other trophoblastic tumors (23). The results of our study indicated that chemotherapy did not significantly affect the prognosis or survival rates of patients diagnosed with PSTT and ETT. Similar studies reinforce this finding, suggesting that chemotherapy should only be considered for high-risk cases (such as advanced-stage or potential recurrence) (1, 13, 20, 24, 25). A specific study at the Women & Infants Hospital-Rhode Island Hospital revealed discouraging outcomes for five patients with PSTT who underwent chemotherapy. While some response was observed, it was short-lived, prompting the suggestion that chemotherapy should be reserved for recurrence after initial surgery, rather than as the first-line treatment (25). Another study showed poor clinical outcomes in patients treated with multiagent chemotherapy, with or without surgery, as 11 of them did not survive (18). However, for women diagnosed with metastatic PSTT/ETT, surgery alone is not curative, necessitating multiagent systemic chemotherapy (6). Initially thought to be unresponsive to chemotherapy, various reports have demonstrated the success of different multiagent chemotherapy approaches in treating metastatic disease (19, 26, 27). Since 75% of the patients included in our study took chemotherapy and the results showed that taking chemotherapy had no significant impact on survival, the idea of whether or not to give chemotherapy to future patients should be considered since chemotherapy treatment is related to many side effects and giving it can do more harm than good. One study outlined several adverse effects in gynecological cancer patients receiving chemotherapy, such as limb numbness, fatigue, hair loss, decreased appetite, taste alterations, muscle pain, nausea, vomiting, itching, or rash (28). Additionally, another study highlighted how cancer and chemotherapy can reduce a patient’s physical activity, alter their appearance, diminish their sense of attractiveness, and subsequently lower their self-esteem (29, 30). Factors such as multiple prior chemotherapy treatments and younger age were identified as predictors of reduced quality of life during chemotherapy in another study (31). Moreover, numerous studies have emphasized the impact of cancer treatment, particularly chemotherapy, on aspects like sexual desire, functioning, and emotional relationships in women (32, 33). Premature menopause resulting from these treatments has been associated with lower quality of life, decreased sexual functioning, distressing menopausal symptoms, psychological distress related to fertility concerns, and uncertainty about the long-term effects of premature menopause (33). Furthermore, a study involving breast cancer patients undergoing chemotherapy reported that 12.50% and 1.78% of participants experienced “moderate” and “severe” depression, respectively (34).

GCC is a rare but highly aggressive form of cancer originating in the trophoblastic cells of the placenta during pregnancy (35, 36). GCC is characterized by rapid growth and a high potential for metastasis, frequently spreading to organs such as the lungs (35, 37). Our study demonstrated that metastases significantly affect OS and CSS. Treatment for GCC typically involves multiagent chemotherapy (38, 39). Although surgery and radiotherapy are used less frequently, they may be necessary for cases of resistant or recurrent GCC (36–38). According to our univariate analysis, chemotherapy did not significantly affect the prognosis or survival rates of patients diagnosed with GCC. While surgery and radiotherapy were significant factors in OS and CSS in the univariate analysis, however, multivariate analysis indicated that surgery and radiotherapy did not significantly affect OS and CSS.

In our study, 31 patients underwent radiotherapy, revealing a negative impact on patient survival, indicating that radiotherapy is an unfavorable prognostic factor. This could be attributed to the physical and psychological side effects of radiation therapy, which substantially influence a patient’s daily functioning and diminish their overall quality of life (40). Yet, it’s important to highlight that one study indicated the potential effectiveness of radiotherapy when applied selectively (19), and another mentioned that two patients achieved complete remission due to pelvic radiotherapy (21).

This study had several limitations that should be considered. The absence of FIGO staging for GTN restricts the depth of the prognostic analysis. Additionally, the retrospective nature of this study introduces inherent limitations as it is not possible to establish causal relationships through retrospective analyses. The absence of specific chemotherapy regimens and surgical procedures limited the ability of this study to provide detailed insights into the efficacy of different therapeutic interventions. Additionally, the use of existing databases limited our ability to stratify indicators, such as the interval between surgery and diagnosis or the site of metastasis, and the rarity of GTN prevented model validation with actual hospital data.

However, this study had several strengths. The incorporation of ML techniques for predicting survival shows the growing potential of AI in healthcare and contributes to the development of personalized medicine. Moreover, this study adopted a comprehensive approach by incorporating a wide range of patient characteristics, tumor features, and treatment options, which provides a holistic understanding of the demographic and clinical aspects of GTN. Furthermore, a large sample size enhances the reliability of the analyses and comparisons, leading to more robust and generalizable results.

## 5 Conclusion

Our research on GTN sheds light on the crucial aspects of clinical decision making, emphasizing the significance of key factors affecting patient outcomes and offering hope for personalized treatment. The cautious use of chemotherapy and radiotherapy is needed because of their potential disadvantages. Further research should explore the molecular markers and validate the generalizability of our findings.

## Data Availability

The original contributions presented in the study are included in the article/**Supplementary Material**. Further inquiries can be directed to the corresponding author.
