# Supplementary material for: Machine learning-driven survival prediction in gestational trophoblastic neoplasms: a focus on PSTT and ETT prognosis
Source: Front Oncol. 2024 Sep 30;14:1457531. doi: 10.3389/fonc.2024.1457531 (PMC11471447; doi:10.3389/fonc.2024.1457531)
Supplement: Supplementary file 1 [file DataSheet1.docx]

| **Years survival** | **GCC** | **ETT** | **PSTT** | **P-value** |
| --- | --- | --- | --- | --- |
| **Overall survival** | | | | |
| 1-year | 0.952  [CI:0.933-0.972] | 0.97  [CI:0.9415-0.9994] | 0.942  [CI:0.898-0.988] | 0.6 |
| 3-year | 0.9331  [CI:0.9102-0.9566] | 0.9229  [CI:0.8781-0.970] | 0.875  [CI:0.8115-0.9443] | 0.4 |
| 5-year | 0.9166  [CI:0.8908-0.9433] | 0.8952  [CI:0.8426-0.9511] | 0.8759  [CI:0.8115-0.9443] | 0.3 |
| **Cancer specific survival** | | | | |
| 1-year | 0.965  [ CI:0.948-0.982] | 1 [CI:1-1] | 0.942  [CI:0.898-0.988] | 0.9 |
| 3-year | 0.9477  [CI:0.9271-0.9688] | 0.9748  [CI:0.9470-1] | 0.909  [CI:0.854-0.968] | 0.4 |
| 5-year | 0.9394  [CI:0.9169-0.9624] | 0.9455  [CI:0.9040-0.9890] | 0.909  [CI:0.854-0.968] | 0.4 |

**Table S1:** Survival rates compared for GCC, ETT, PSTT over 1, 3, 5 years.

**Table S2:** Machine learning algorithms performance

| Cancer type | ML Algorithm | 10-fold Cross Validation | Accuracy | Mean Bootstrap Estimate [95% CI] | AUC |
| --- | --- | --- | --- | --- | --- |
| GCC | LR | 0.708 | 0.66 | 0.67 [0.58–0.76] | 0.75 |
|  | KNN | 0.814 | 0.65 | 0.64 [0.54–0.74] | 0.877 |
|  | RFC | 0.816 | 0.77 | 0.79 [0.69–0.89] | 0.877 |
|  | GBC | 0.781 | 0.83 | 0.80 [0.69–0.90] | 0.904 |
|  | MLP | 0.722 | 0.67 | 0.71 [0.60–0.81] | 0.745 |
|  |  |  |  |  |  |
| PSTT + ETT | LR | 0.737 | 0.70 | 0.61 [0.46–0.73] | 0.746 |
|  | KNN | 0.670 | 0.70 | 0.60 [0.46–0.74] | 0.805 |
|  | RFC | 0.812 | 0.76 | 0.68 [0.55–0.81] | 0.805 |
|  | GBC | 0.740 | 0.72 | 0.71 [0.58–0.86] | 0.803 |
|  | MLP | 0.720 | 0.76 | 0.63 [0.51–0.76] | 0.822 |

**
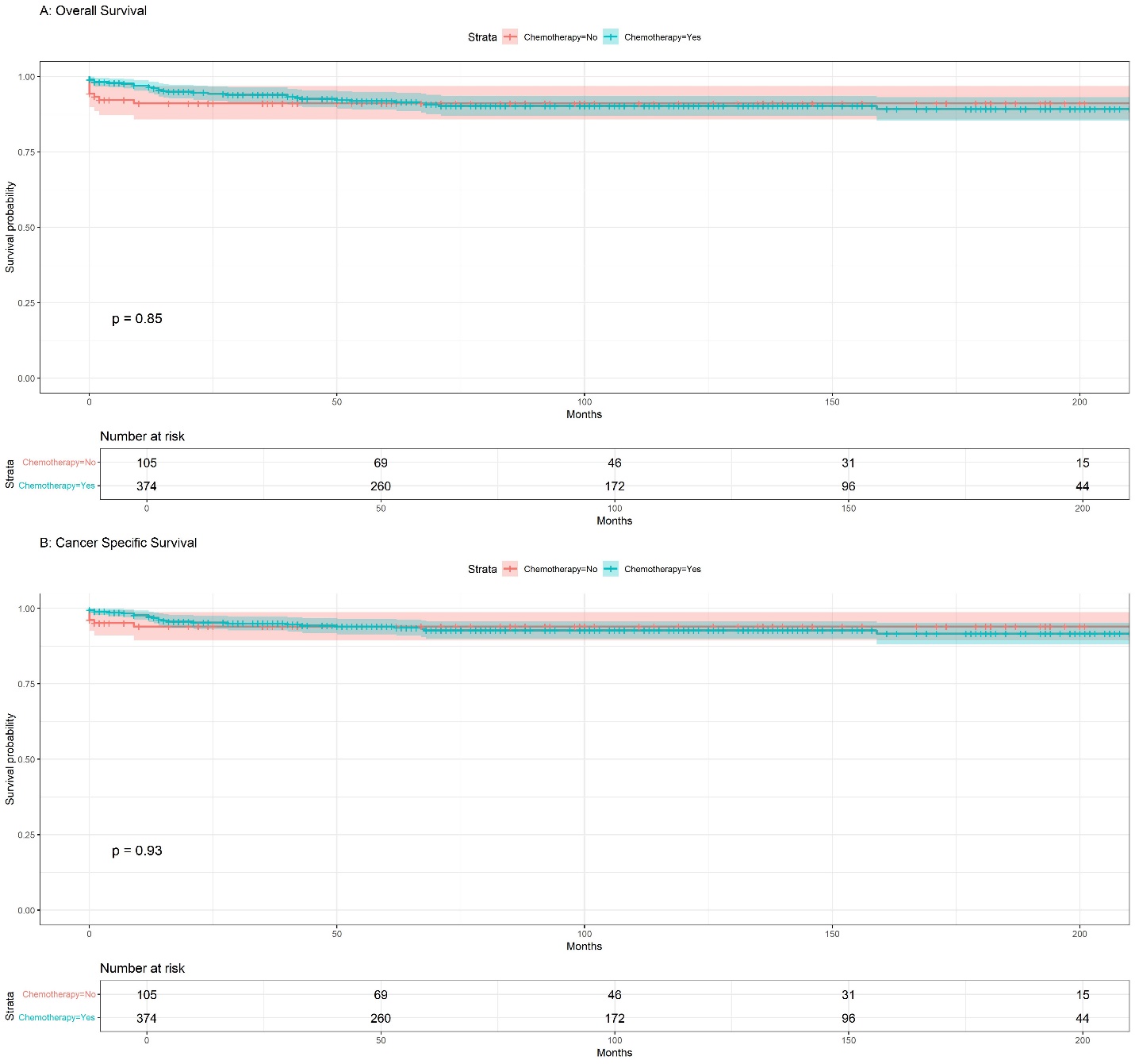
**

**FigureS1 -GCC: a)** Kaplan-Meier overall survival (OS) based on chemotherapy; **b)** Kaplan-Meier cancer-specific survival (CSS) based on chemotherapy.

**
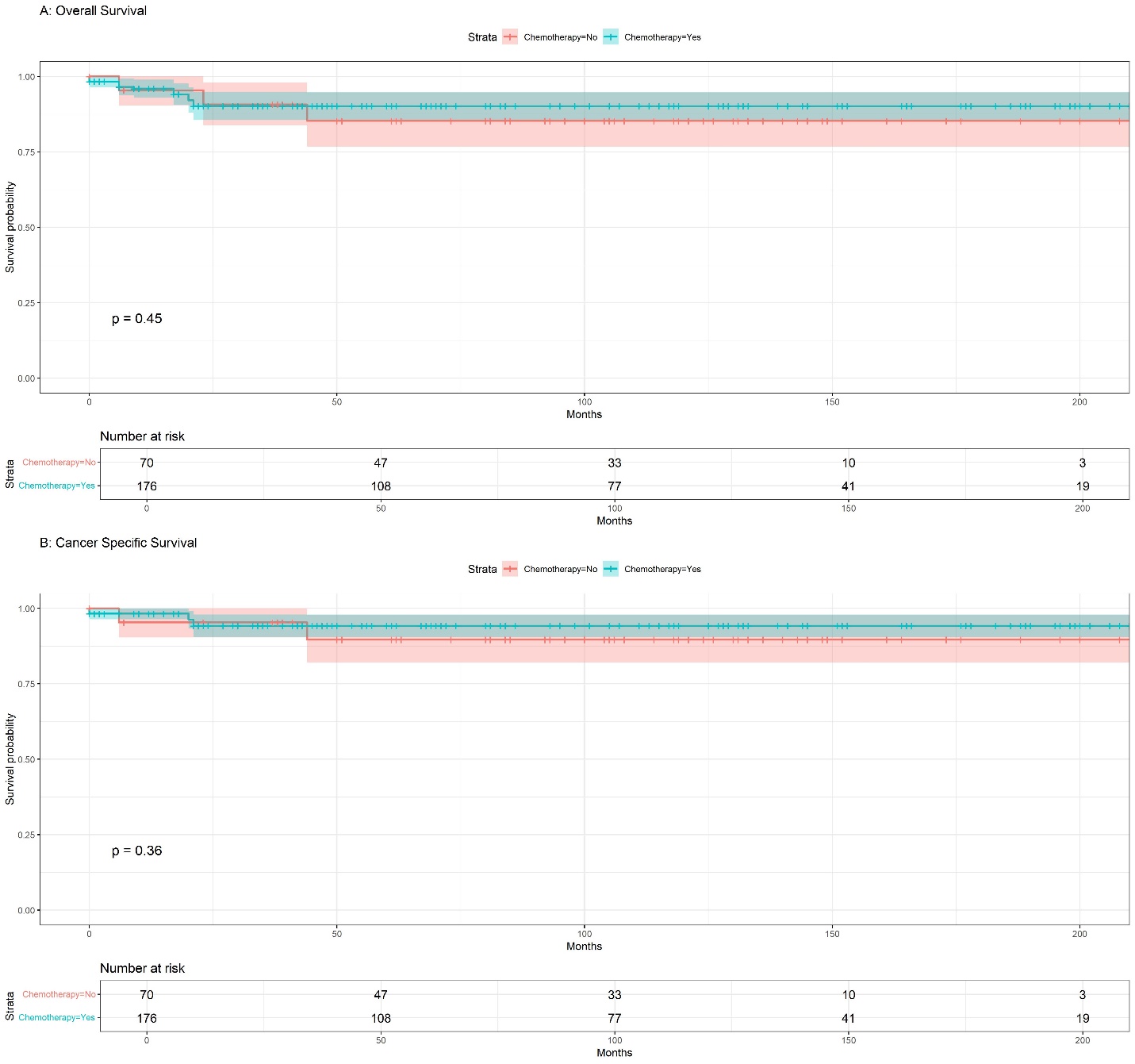
**

**Figure S2-PTSS+ETT: a)** Kaplan-Meier overall survival (OS) based on chemotherapy; **b)** Kaplan-Meier cancer-specific survival (CSS) based on chemotherapy.
